# Population, Reproductive, and Sexual Health: Data Are Essential Where Disciplines Meet and Ideologies Conflict

**DOI:** 10.3389/fpubh.2016.00027

**Published:** 2016-03-07

**Authors:** Joseph B. Stanford

**Affiliations:** ^1^Department of Family and Preventive Medicine, University of Utah School of Medicine, Salt Lake City, UT, USA; ^2^Department of Obstetrics and Gynecology, University of Utah School of Medicine, Salt Lake City, UT, USA; ^3^Department of Pediatrics, University of Utah School of Medicine, Salt Lake City, UT, USA

**Keywords:** reproductive health, population dynamics, sexual health, oppositional collaboration, adolescent pregnancy

The flourishing of the individual human person, the health of human society, and the ecological well being of planet earth are inextricably connected with the issues of human population, sexuality, and reproduction. Many academic disciplines have strong interests in these issues, approaching them from different perspectives and with different emphases. However, these areas of study are also infused with controversy and strong ideological positions arising from cultural, religious, political, and social traditions that sometimes clash with each other. To address these issues, two things are needed: (1) data that address questions from different underlying assumptions; (2) open and respectful discussion among scientists, clinicians, and policy makers who have different backgrounds, narrative frameworks, and conceptual perspectives (1). The new Section on Population, Reproductive and Sexual Health, Frontiers in Public Health will contribute constructively to these critical needs.

There is no substitute for data, carefully collected, analyzed, and considered, to contribute to and inform scientific and policy discussion in healthy, transformative ways. It is universal to human nature that preconceived assumptions drive perceptions and explanatory models: ultimately one can only see what one is willing to consider might be true. Scientists are not exempt from cognitive bias (2, 3). However, the essence of the scientific method is that data are allowed to challenge assumptions. Scientists and professionals have the great opportunity to allow their models of the world to be influenced, updated, and improved by data, carefully collected and objectively analyzed.

Research results can and should fundamentally inform theory and challenge assumptions, regardless of their popularity or social currency within the researchers’ own peer groups. Consider as an example, research that has challenged both advocates and opponents of emergency (postcoital) contraception (4). Advocates for emergency contraception have proposed confidently that widespread dissemination and promotion of emergency contraception would decrease rates of unintended pregnancy and induced abortion (5, 6). However, the large preponderance of evidence from extensive interventional research is that emergency contraception has not decreased unintended pregnancy nor induced abortion, and is unlikely to do so (7–10). Additionally, levonorgestrel emergency contraception is certainly much less effective to prevent pregnancy than originally proposed (11–13). On the other hand, skeptics and opponents of emergency contraception have assumed or proposed that levonorgestrel emergency contraception acts after fertilization to prevent successful implantation of the embryo (14–16). However, the preponderance of recent evidence for postcoitally administered levonorgestrel does not support this as a significant mode of action (17, 18), although some gaps in data and differences in interpretation remain to be explored (19, 20).

Science does not give us human or ethical values nor does it weigh the relative importance of different questions. Scientists coming from different worldviews will ask very different questions. If some questions are not asked, it is unlikely that data will be collected to answer them. It follows that there is great value in a spectrum of research conducted by scientists with an array of different backgrounds and world views. For example, in relation to infertility, one set of questions can be summarized: how can methods of *in vitro* fertilization be improved for better outcomes for couples with subfertility? (21, 22). In contrast, another set of questions can be summarized: how can underlying causes of infertility be identified to prevent subfertility (23), or corrected or improved to increase the chances of *in vivo* conception for couples with subfertility? (24, 25). These different types of questions will generate very different kinds of research, results of which will advance human understanding in different dimensions.

This section will welcome research addressing well formulated questions from all perspectives, including questions that may be controversial or challenge current paradigms. Questions such as what are the health sequelae of elective abortion? (26, 27), under what circumstances may promotion of different types of contraception decrease or increase the incidence of elective abortion? (28, 29), what are positive or negative consequences of contraceptive policy that privileges long-acting reversible contraceptives? (30, 31), what are positive or negative consequences of a wider dissemination of natural family planning methods based on fertility awareness? (32–34), how can women and men more readily understand their mutual fertility? (35), which of the many new mobile apps and devices provide reliable data to women about the fertile window? (36), what is the impact of sexual activity on the timing of ovulation? (37), what types of school-based interventions reduce rates of sexually transmitted diseases or pregnancy in different social contexts? (38–41), are demographic transitions leading to overpopulation or eventual underpopulation? (42), what is the intergenerational health impact of parental health, and of different types of fertility treatment? (43–45), what are the impacts of environmental exposures on reproductive health? (46, 47), is human fecundity decreasing? (48, 49), and what is the link between human fecundity and other dimensions of human health? (50, 51).

The foregoing is not intended as a comprehensive or representative list of the topics suitable for the Section on Population, Reproductive and Sexual Health. It is intended to stimulate thought about the extensive possibilities and needs for interdisciplinary research. No doubt readers will come up with many other important questions. For all questions and perspectives in population, reproductive, and sexual health, methodologically sound research is welcome at this section.

I expect that some of the best work in the future for population, reproductive, and sexual health will be accomplished by scientists with very different underlying assumptions or ideologies who find ways to work together. A contemporary philosopher has suggested a paradigm of “oppositional collaboration” for areas with high ideological polarization, in which bioethicists (or scientists) with fundamentally opposed viewpoints work together to generate data that they all agree is as objective as possible for the relevant questions. This does not necessarily result in a change in values or agreement of the respective colleagues, but it can result in more accurate data and increased understanding and respect, extremely valuable outcomes (52). I personally have found that research is often more fruitful when scientists with different underlying values and ideologies work together with a common commitment to obtaining objective data (53, 54). I commend this approach for consideration by researchers submitting to this section.

We are committed to fair review and rapid dissemination of carefully conducted science in population, reproductive, and sexual health. We are particularly interested in research that asks questions that may be neglected in this field and research that can facilitate data-based dialogue across disciplines and ideologies. These goals are supported by the innovative Frontiers model for scientific publishing, which includes a large editorial review board and distributed editorial independence (i.e., publication decisions are made primarily at the level of the associate editor), enhanced interaction between the review editors and authors, open-access publishing, and a robust infrastructure for post-publication professional discussion. We look forward to new research contributions and the associated discussions.

## Author Contributions

The contribution is the sole responsibility of the single author.

## Conflict of Interest Statement

The author declares that the research was conducted in the absence of any commercial or financial relationships that could be construed as a potential conflict of interest.
